# Case report: Challenging kidney transplantation in an adolescent patient with tetralogy of Fallot

**DOI:** 10.3389/fmed.2024.1327363

**Published:** 2024-07-10

**Authors:** Ante Jakšić, Berislav Barbalić, Lidija Orlić, Željko Župan, Božidar Vujičić, Antun Gršković, Tanja Ćelić, Ivana Koraca Chinchella, Neven Čače, Sanja Flajšman-Raspor, Ivan Bubić, Josip Španjol, Dean Markić

**Affiliations:** ^1^Department of Urology, Clinical Hospital Center Rijeka, Rijeka, Croatia; ^2^Faculty of Medicine, University of Rijeka, Rijeka, Croatia; ^3^Department of Anesthesiology and Intensive Care, University Hospital Rijeka, Rijeka, Croatia; ^4^Department of Nephrology, Dialysis and Transplantation, University Hospital Rijeka, Rijeka, Croatia; ^5^Department of Pediatrics, University Hospital Rijeka, Rijeka, Croatia

**Keywords:** cardio-renal syndrome, congenital heart disease, chronic renal insufficiency, kidney transplantation, tetralogy of Fallot

## Abstract

Tetralogy of Fallot is the most common cyanotic congenital heart disease. This severe disorder of cardiac physiology can impair renal function and lead to the development of cardiorenal syndrome and eventually to end-stage renal disease. Kidney transplantation may be the best option for renal replacement treatment in patients with tetralogy of Fallot, but only after correcting cardiac abnormalities and optimizing cardiac functions, all of which require a multidisciplinary approach. We report the first case of kidney transplantation in an adolescent patient with tetralogy of Fallot. Our findings confirms that kidney transplantation is a valuable treatment option in selected congenital heart disease cases.

## Introduction

Tetralogy of Fallot (ToF) is the most common cyanotic congenital heart disease (CHD) (1). It is characterised by a large ventricular septal defect, overriding aorta, right ventricular outflow tract (RVOT) obstruction with pulmonary valve stenosis, and right ventricular hypertrophy. The right-to-left shunt decreases the oxygen saturation in the systemic circulation and decreases tissue oxygenation, resulting in cyanosis. The overall survival rate is low without surgical treatment, and more than 90% of patients die before the age of 40 (2).

Renal dysfunction is a common consequence of CHD, known as cyanotic nephropathy (3, 4). We present the peculiar case of successful kidney transplantation (KT) in a patient with ToF and consequent chronic kidney disease (CKD).

## Case report

A 16-year-old female with end-stage renal disease (ESRD) was admitted to our hospital for kidney transplantation. She was diagnosed with ToF, which caused cyanotic nephropathy, leading to ESRD over the years.

**Figure 1:**
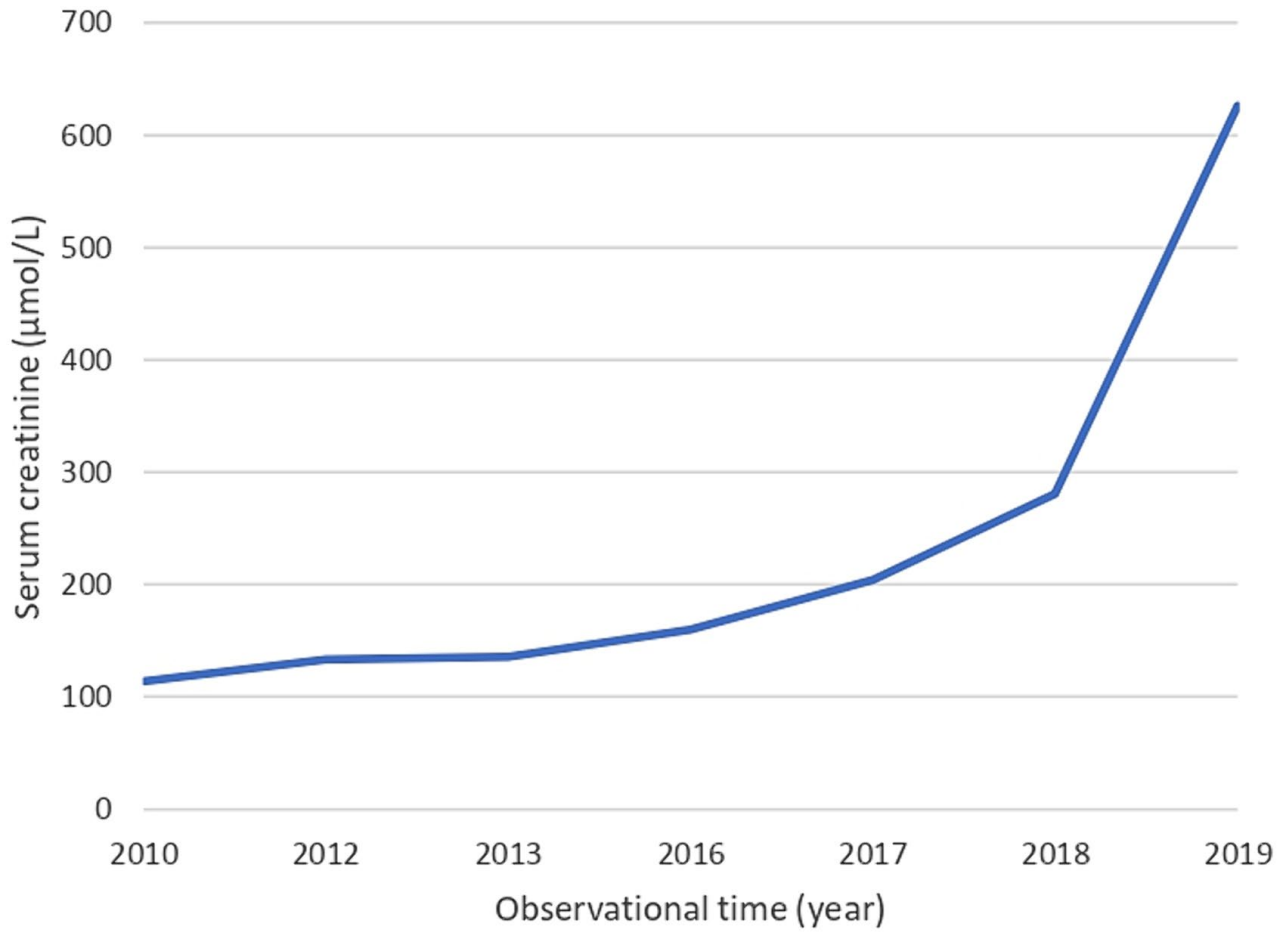
Serum creatinine values over the years. The blue line illustrates the changes in creatinine levels over the years until the beginning of hemodialysis.

The patient had sudden cyanosis immediately after birth. Transthoracic echocardiography and cardiovascular magnetic resonance imaging confirmed the diagnosis of ToF. At three weeks of age, the patient underwent the first surgery to create a central aortopulmonary shunt. Due to CHD and heart surgery, acute kidney injury (AKI) developed and required renal replacement treatment. She was on continuous ambulatory peritoneal dialysis (CAPD) from November 2005 to April 2007. In June 2007, the cardiac team extended and dilated both branches of the pulmonary artery and the main pulmonary artery, reconstructed the pulmonary valve, and closed the ventricular septal defect. Progress in growth and development has been noticeable since the second heart surgery. Renal function recovered and the peritoneal catheter was removed in September 2007. From 2007 to 2019 she was regularly followed by pediatric cardiologists, nephrologists, and neurologists. Therapy regimen included enalapril twice daily in two divided doses (2 mg and 1.25 mg), atenolol 25 mg once daily, alopurinol 100 mg once daily, calcium carbonate 500 mg once daily, and calcitrol 0.25 mg every other day. Urine output was unchanged, up to 3 L/day, with normal fluid intake. Although renal function had slowly deteriorated over the years, the patient did not require renal replacement treatment, and no episodes of AKI occurred (Figure 1). However, at the age of 14 years, there was a sudden deterioration in renal function and cardiac status, including severe pulmonary insufficiency and RVOT pseudoaneurysm. Through interdisciplinary collaboration, we put her on the transplant waiting list and referred her for a third heart surgery. The cardiac team reconstructed the right ventricular outflow tract, replaced the pulmonary valve, and dilated the left branch of the pulmonary artery. In February 2020, at the age of 15, she started hemodialysis (HD). Considering the patient’s age and side effects of HD, we decided KT was the best treatment option for our patient.

**Figure 2:**
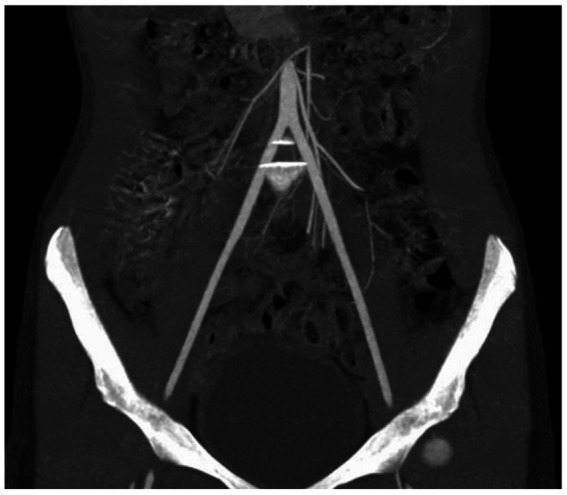
Preoperative multislice computerized tomography scan of the external iliac arteries. Representative coronal image of patient’s CT scan of the external iliac arteries with contrast performed on the day of surgery, illustrating gracile external iliac arteries with a slightly larger diameter of the left external iliac artery.

In December 2021, the patient received a cadaveric kidney transplant. During the pretransplant evaluation, we used magnetic resonance aortography to measure the sizes of the iliac blood vessels. The left external iliac artery had a diameter of 6 mm, slightly larger than the right external iliac artery, which measured 5.3 mm (Figure 2). The cardiological examination included transthoracic echocardiography and electrocardiogram (ECG). There was no right ventricular dilatation, ventricular shunt, or pulmonary hypertension. Systolic function was good with an ejection fraction of 40%. The ECG was normal except for a right bundle branch block. Her New York Heart Association (NYHA) functional classification was I, with a BMI of 15.6 kg/m^2^, ASA 3, and MET<4. Before surgery, she received induction therapy (basiliximab 20 mg) followed by standard triple immunosuppressive therapy of tacrolimus with trough levels of 8 ng/mL, mycophenolate mofetil 500 mg twice daily, and methylprednisolone 250 mg once daily, as well as prophylactic treatment with teicoplanin 250 mg once daily and meropenem 500 mg three times daily. A PiCCO (Pulse index Continuous Cardiac Output) catheter was inserted during the procedure for minimally invasive hemodynamic monitoring. The kidney was allocated through Eurotransplant, and the donor was a 36-year-old brain-dead male with a ruptured cerebral aneurysm. Crossmatch (Luminex) was negative, with good HLA matching (5/10). Due to the recipient’s small external iliac vessels’ diameter, we used a left pararectal incision and anastomosed the donor renal artery and vein end-to-side with the common iliac vessels. The vascular anastomosis time was 35 min, the cold ischemia time was 15 h, and the overall surgical time was 120 min.

On the first postoperative day, graft performance was excellent, and creatinine decreased from 467 μmol/L before surgery to 275 μmol/L the following day and normalised on the fifth day. The echocardiogram remained unchanged after KT. The patient had no post-transplant surgical or cardiac complications and was discharged after three weeks. Double J stent was removed 6 weeks after the transplantation. Fourteen months after surgery, the patient had a fully functional allograft with a creatinine level of 79 μmol/L. Ultrasound examination showed good vascularisation of the entire allograft, with a resistance index between 0.52–0.54. In addition, heart function remained stable after KT. To date, kidney function and cardiac status remained normal.

## Discussion

Patients with ToF and other CHD are at high risk for CKD (4–8). Previous studies stressed the importance of recognizing cyanotic nephropathy early on, as it is strongly associated with progressive CKD. Screening patients with CHD for significant microalbuminuria may be a useful strategy for early detection of CKD (4). CKD can develop in cyanotic CHD patients during childhood or adolescence (6, 7). Fang et al. showed that 167/359 (46.5%) patients with CHD developed CKD during follow-up (6). The mean age at the onset of CKD was 14 years, as in our case.

CKD in patients with ToF probably has multiple contributing factors. Novel pathophysiological postulates of CKD development in cyanotic CHD patients include the effects of chronic direct hypoxia and reactive erythrocytosis on glomerular capillary damage. Erythrocytosis induces secondary hyperviscosity of the blood and eventually leads to glomerulosclerosis (9). Cyanosis also damages the proximal convoluted tubules and causes interstitial fibrosis (10). Kidney injury can also occur from the use of contrast media for diagnostic tests and nephrotoxic drugs (4–6). Moreover, previous cardiac surgery significantly raises the risk of kidney injury in patients with ToF and other cyanotic CHD (11–13). ESRD is likely to be multifactorial in our patient, given the history of cyanotic nephropathy, three cardiac surgeries, use of loop diuretics, and exposure to contrast.

There are literature limitations regarding the treatment of ESRD in patients with ToF and CHD in general (14–16). To date, the best option for renal replacement treatment in this population remains a matter of debate. Furthermore, it requires a cautious approach due to potential complications. HD can lead to sepsis, pulmonary embolism, infective endocarditis, and heart failure via peripheral vascular access (15). On the other hand, PD solution infusion increases abdominal pressure and diaphragm elevations during PD, which can lead to potentially fatal respiratory failure, especially in children. Contrary to HD, PD has minimal impact on the intravascular volume status, reduces cardiovascular stress, avoids peaks and troughs in uremic toxins, and has a better preservation of residual renal function (16). Delayed KT can cause severe anemia and mineral-bone disorders, leading to vascular calcification and potential cardiovascular complications, impaired linear growth, and delayed sexual maturity (17). It is known that KT improves cognitive development, skeletal growth, health-related quality of life, and overall survival in children with ESRD compared to dialysis (18). However, KT recipients with CHD have a lot of non-modifiable comorbidities caused by pre-existing heart issues and past treatments which are difficult to treat during the transplantation. Consequently, the post-transplant period becomes more complex to treat (19).

Preoperative planning of KT in ToF patients and other CHD population, must focus on two aspects beyond standard investigations. First, as in adults, an assessment of the iliac vessels for vascular anastomosis using MSCT or magnetic resonance aortography. The second aspect is cardiac status. Correction of cardiac abnormalities is mandatory before KT as it prevents the recurrence of cyanotic nephropathy and avoids additional cardiac surgery with possible adverse effects on renal function (11, 19).

KT is a valuable option for the pediatric kidney transplant population since it has significantly reduced mortality and improved their quality of life (17, 18, 20). Currently, there are no data on KT in patients with ToF. To the best of our knowledge, this is the first case of KT in a ToF patient with CKD.

## Conclusion

Tetralogy of Fallot is a severe congenital heart disease. Due to the development of cardiorenal syndrome, it can lead to end-stage renal disease. Kidney transplantation seems to be a feasible treatment option for patients with tetralogy of Fallot and end-stage renal disease, as shown in our case. Correct preoperative assessment and selection of the patients through a multidisciplinary approach is mandatory. The promising results reported in this case warrant further studies on kidney transplantation in patients with cyanotic congenital heart disease.

## Data availability statement

The raw data supporting the conclusions of this article will be made available by the authors, without undue reservation.

## Ethics statement

The studies involving humans were approved by Ethics Committee of the Clinical Hospital Center Rijeka. The studies were conducted in accordance with the local legislation and institutional requirements. Written informed consent for participation in this study was provided by the participants’ legal guardians/next of kin. Written informed consent was obtained from the minor(s)’ legal guardian/next of kin for the publication of any potentially identifiable images or data included in this article. Written informed consent was obtained from the participant/patient(s) for the publication of this case report.

## Author contributions

AJ: Conceptualization, Data curation, Investigation, Methodology, Visualization, Writing – original draft, Writing – review & editing. BB: Investigation, Writing – review & editing. LO: Conceptualization, Supervision, Writing – review & editing. ŽŽ: Conceptualization, Supervision, Writing – review & editing. BV: Data curation, Writing – review & editing. AG: Conceptualization, Methodology, Writing – review & editing. TĆ: Methodology, Supervision, Writing – review & editing. IC: Investigation, Writing – review & editing. NČ: Investigation, Writing – review & editing. SF-R: Investigation, Writing – review & editing. IB: Investigation, Methodology, Writing – review & editing. JŠ: Investigation, Supervision, Validation, Writing – review & editing. DM: Data curation, Investigation, Methodology, Supervision, Validation, Writing – review & editing.
